# Prior hospitalizations as a predictor of prognosis in heart failure with mildly reduced ejection fraction

**DOI:** 10.1007/s00392-025-02612-9

**Published:** 2025-02-18

**Authors:** Henning Johann Steffen, Michael Behnes, Alexander Schmitt, Noah Abel, Felix Lau, Marielen Reinhardt, Muharrem Akin, Thomas Bertsch, Mohamed Ayoub, Kambis Mashayekhi, Kathrin Weidner, Ibrahim Akin, Tobias Schupp

**Affiliations:** 1 Department of Cardiology, Angiology, Haemostaseology and Medical Intensive Care, First Department of Medicine, Medical Faculty Mannheim, University Medical Centre Mannheim, Heidelberg University, Theodor-Kutzer-Ufer 1-3, 68167 Mannheim, Germany; 2 Department of Cardiology, St. Josef-Hospital, Ruhr-Universität Bochum, 44791 Bochum, Germany; 3 Institute of Clinical Chemistry, Laboratory Medicine and Transfusion Medicine, Nuremberg General Hospital, Paracelsus Medical University, 90419 Nuremberg, Germany; 4 Division of Cardiology and Angiology, Heart Center University of Bochum, Bad Oeynhausen, Germany; 5 Department of Internal Medicine and Cardiology, Mediclin Heart Centre Lahr, Lahr, Germany

**Keywords:** Heart failure with mildly reduced ejection fraction, HFmrEF, Heart failure, Prior hospitalizations, Prognosis

## Abstract

**Objective:**

This study aims to investigate the prognostic impact of the presence and type of prior hospitalizations in patients with heart failure with mildly reduced ejection fraction (HFmrEF).

**Background:**

Data investigating the prognostic impact of the present and type of previous all-cause hospitalizations in HFmrEF is limited.

**Methods:**

Consecutive patients hospitalized with HFmrEF at a single medical center were retrospectively included from 2016 to 2022. The prognosis of patients with a prior hospitalization < 12 months was compared to patients without. The primary endpoint was all-cause mortality at 30 months (median follow-up), the key secondary endpoint was heart failure (HF)-related rehospitalization at 30 months.

**Results:**

Two thousand one hundred eighty four patients with HFmrEF were included, 34.8% had a previous hospitalization < 12 months (admission to internal medicine and geriatrics: 60.8%, surgical department: 23.5%). The presence of a previous hospitalization was associated with an increased risk of all-cause mortality (38.6% vs. 27.4%; HR = 1.51; 95% CI 1.30–1.76; *p* = 0.01) and HF-related rehospitalization at 30 months (21.2% vs. 9.1%; HR = 2.48; 95% CI 1.96–3.14; *p* = 0.01), even after multivariable adjustments. However, the department of previous hospitalization (internal medicine vs. surgical) did not significantly affect the risk of 30-months all-cause mortality (37.1% vs. 43.2%; HR = 0.82, 95% CI 0.63–1.08; *p* = 0.16) or HF-related rehospitalization (24.0% vs. 16.8%; HR = 1.47, 95% CI 0.98–2.24; *p* = 0.07). Finally, the type of previous admission (i.e., elective, emergency vs. HF-related admission) (log-rank *p* = 0.29) did not affect the risk of 30-months all-cause mortality.

**Conclusion:**

Prior hospitalizations within 12 months were independently associated with impaired long-term mortality in patients with HFmrEF, irrespective of the department or type of prior admission.

## Introduction

In developed countries, novel evidence-based treatments have decreased the age-adjusted incidence of heart failure (HF), however, the overall incidence of HF is continuously increasing related to an improving life-expectancy [1–5]. In 2016, the European Society of Cardiology (ESC) HF guidelines introduced HF with mildly reduced ejection fraction (i.e., HFmrEF; left ventricular ejection fraction (LVEF) 41–49%) as a new HF subgroup with “intermediate” characteristics compared to HF with preserved (i.e., HFpEF) and reduced LVEF (i.e., HFrEF) [5–8]. Besides its association with an overall increased all-cause mortality, HF in general is a prevalent cause of recurrent hospitalizations. A higher risk of mortality in patients with more frequent HF readmissions with a 1-year mortality rate of up to 27% in patients with two HF hospitalizations was specifically observed in “lower risk” HF inpatients [9]. From this perspective, HF-related and all-cause readmissions at 30 and 90 days have increased from 2010 to 2017 within a registry-based study including over 6,000,000 HF hospitalizations [10]. In line, a hospitalization < 6 months was shown to be associated with increased mortality risks in both patients with HFrEF or HFpEF [11, 12]. Further studies demonstrated notable differences in all-cause readmission rates between HFrEF and HFpEF. For instance, Tay et al*.* [13] suggested that all-cause readmissions occurred in 25% of HFrEF patients and 20% of HFpEF patients, whereas readmissions in HFpEF were primarily linked to non-HF causes (72%), whereas 45% of HFrEF readmissions were related to HF. Cheng et al. [14] indicated that both HFpEF and HFmrEF had lower mortality and higher all-cause readmission risks than HFrEF, although these differences did not persist after multivariable adjustment. These findings suggest that patients with HFmrEF, may benefit from a management focus on non-cardiovascular comorbidities to reduce all-cause readmissions, which may be comparable to patients with HFpEF.

When comparing different HF categories, early readmissions in HF may be frequently driven by non-cardiovascular causes, especially in HFpEF compared to HFrEF [15]. Specifically non-cardiac comorbidities, particularly chronic kidney disease (CKD), anemia, and pulmonary hypertension (PH), as primary drivers of non-cardiac rehospitalization in HF patients, may deteriorate prognosis by both the presence and management of these conditions [13]. Despite the “intermediate” characteristics of patients with HFmrEF, the burden of non-cardiac comorbidities may be higher in HFmrEF compared to HFrEF, demanding the need to further investigate the prognostic value of different types of admissions (cardiac vs. non-cardiac) in this population [16].

Therefore, the aim of the present study was to investigate the prognostic impact of a prior hospitalization < 12 months including a large-scaled registry of patients hospitalized with HFmrEF. Furthermore, the prognostic impact of the admission department (i.e., internal medicine vs. surgical) and type (i.e., elective vs. emergency vs. HF-related) of prior hospitalization was investigated.

## Methods

### Study patients, design and data collection

For the present study, consecutive patients hospitalized with HFmrEF were included at a single institution from January 2016 to December 2022 [17]. Utilizing the electronic hospital information system, we systematically gathered comprehensive clinical data pertaining to the index event, including baseline characteristics, admission vital signs, medical history, prior treatments, duration of hospital and intensive care unit (ICU) stay, laboratory values, and details from non-invasive or invasive cardiac diagnostics and device therapies (e.g., echocardiography, coronary angiography, and information from cardiac devices).

This study originated from the “Heart Failure with Mildly Reduced Ejection Fraction Registry” (HARMER), a retrospective single-center registry comprising consecutive HFmrEF patients admitted to the University Medical Center Mannheim (UMM), Germany. (clinicaltrials.gov identifier NCT05603390). The registry was carried out in accordance to the Declaration of Helsinki and approval from the Medical Ethics Committee II of the Medical Faculty Mannheim, University of Heidelberg, Germany (ethical approval code: 2022-818).

### Inclusion and exclusion criteria

All individuals aged 18 years or older, hospitalized at a single institution with HFmrEF, were retrospectively included in this study. The diagnosis of HFmrEF were established in accordance with the “2021 ESC Guidelines for the diagnosis and treatment of acute and chronic HF” [18]. Patients with a LVEF between 41 and 49%, accompanied by clinical symptoms and/or signs indicative of HF, were included. The presence of elevated amino-terminal prohormone of brain natriuretic peptide (NT-proBNP) levels and other indications of structural heart disease were considered supportive of the diagnosis, but not mandatory for the diagnosis of HFmrEF. Transthoracic echocardiography, performed by cardiologists following current European guidelines [19] during routine clinical care. Echocardiographic operators were blinded to the final study analyses. All source data from echocardiographic examinations, including imaging files and reports, underwent post-hoc reassessment by two independent cardiologists for the purposes of this study. Patients below the age of 18 years were excluded from the study. No additional exclusion criteria were applied.

### Risk stratification

In the present study, patients with at least one prior hospitalization for 12 months of the index hospital admission at our institution were compared to patients. Ambulatory visits at our institution were not considered as a prior hospitalization. In addition, we examined the prognosis of patients with a prior hospitalization < 12 months, distinguishing between those admitted to a department of internal medicine and those admitted to a surgical department (i.e., general surgery, orthopedics, urology). In patients with multiple prior hospitalizations within < 12 months, risk stratification was performed related to the admission department with the closest time interval to the index hospital admission for HFmrEF. Further risk stratification was performed according to the type of prior admission (i.e., prior HF-related admissions, prior elective admissions, and prior emergency admissions not related to HF).

### Study endpoints

The primary endpoint of this study was all-cause mortality during a median follow-up of 30 months. Secondary endpoints included in-hospital all-cause mortality, all-cause mortality at 12 months, rehospitalization for worsening HF, cardiac rehospitalization, acute myocardial infarction (AMI), stroke, coronary revascularization, and major adverse cardiac and cerebrovascular events (MACCE). All-cause mortality data was collected through the electronic hospital information system and direct communication with state resident registration offices (‘bureau of mortality statistics’). Out of an initial cohort of 2228 patients with HFmrEF, 44 individuals with no evidence during long-term follow-up were excluded (i.e., lost-to-follow-up rate of 1.97%). HF-related hospitalization was defined as rehospitalization for HF with the need for intravenous diuretic therapy. Cardiac rehospitalization was specified as rehospitalization primarily attributable to a cardiac condition, including worsening HF, AMI, coronary revascularization, and symptomatic atrial or ventricular arrhythmias. MACCE was defined as a composite endpoint comprising all-cause mortality, coronary revascularization, non-fatal AMI, and non-fatal stroke.

### Statistical methods

Quantitative data is expressed as mean ± standard error of mean (SEM), median with interquartile range (IQR), depending on the distribution of the data. Statistical comparisons were conducted using the Student’s *t* test for data exhibiting normal distribution. The Mann–Whitney *U* test was employed for nonparametric datasets. Normality of distribution was assessed through the Kolmogorov–Smirnov test. Qualitative data is displayed as absolute and relative frequencies, and their comparisons were performed utilizing the Chi-square test or Fisher’s exact test, as deemed appropriate for the specific analytical context. Kaplan–Meier analyses were performed stratified by the presence or absence of prior hospitalization < 12 months, as well as stratified by the department or type of admission of prior hospitalization. Univariable hazard ratios (HR) were given together with 95% confidence intervals. Thereafter multivariable Cox regression analyses were performed investigating the prognostic impact of prior hospitalization < 12 months in patients with HFmrEF. For Cox regression models, univariable Cox regression analyses were performed including characteristics and comorbidities that were yet demonstrated to affect outcomes in HF patients. Only parameters with *p* ≤ 0.10 within univariable Cox regression models were included in multivariable Cox regression analyses.

Results of all statistical tests were considered significant for *p* ≤ 0.05. SPSS (Version 28, IBM, Armonk, New York) was used for all statistical analyses.

## Results

### Study population

**Fig. 1:**
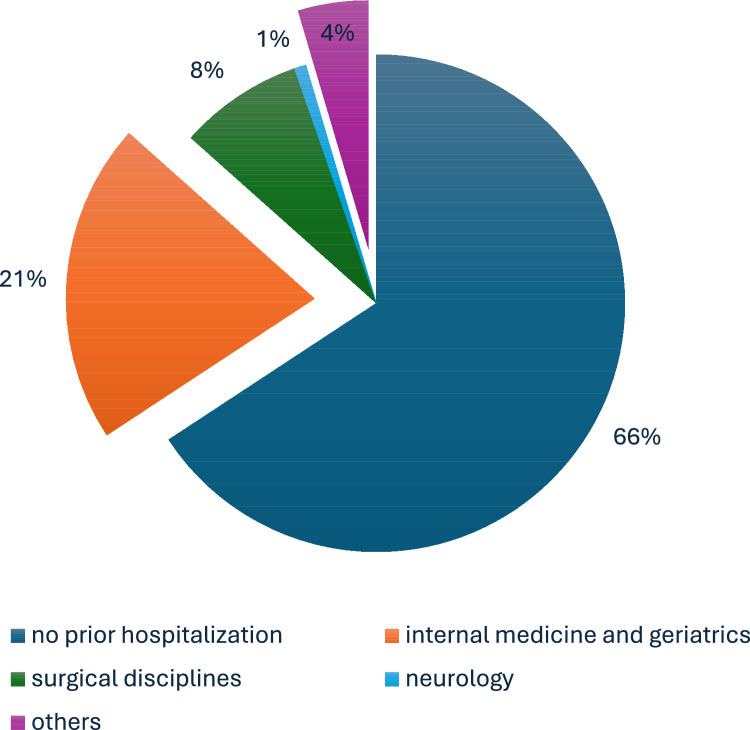
Pie chart illustrating the distribution of admission departments regarding prior hospitalizations < 12 months in patients with HFmrEF

From 2016 to 2022, 2228 patients with HFmrEF were hospitalized at our institution. After excluding 44 patients lost to follow-up, the final study cohort comprised 2184 patients included in the HARMER registry. Of those, 34.8% (*n* = 748) had experienced at least one prior hospitalization < 12 months. Most patients (i.e., 60.8%) (*n* = 455) were previously admitted to a department of internal medicine and geriatrics, 23.5% (*n* = 176) to a surgical department (i.e., surgery, orthopedics, or urology), 2.3% (*n* = 17) to our neurologic department, and 13.4% (*n* = 100) were admitted to other departments, respectively (Fig. 1). Patients with prior hospitalization exhibited a significantly higher prevalence of cardiovascular comorbidities, including higher rates of prior coronary artery disease (CAD) (52.9% vs. 34.8%; *p* = 0.01), arterial hypertension (81.7% vs. 76.0%; *p* = 0.01), prior AMI (31.6% vs. 19.8%; *p* = 0.01), prior percutaneous coronary interventions (PCI) (37.7% vs. 23.0%; *p* = 0.01), prior coronary artery bypass grafting (CABG) (11.9% vs. 8.7%; *p* = 0.02), alongside with higher rates of prior congestive HF (56.7% vs. 22.1%; *p* = 0.01) and decompensated HF < 12 months (31.8% vs. 0.0%; *p* = 0.01) (Table 1). In addition, they had higher rates of concomitant CKD (44.9% vs. 23.9%; *p* = 0.01), peripheral artery disease (17.0% vs. 8.8%; *p* = 0.01), stroke (18.4% vs. 13.4%; *p* = 0.01), malignancies (23.7% vs. 11.0%; *p* = 0.01) and chronic obstructive pulmonary disease (COPD) (16.6% vs. 9.7%; *p* = 0.01).

**Table 1:** Baseline characteristics

	No prior hospitalization (*n* = 1436)		Prior hospitalization (*n* = 748)		*p* value
Age, median (IQR)	76	(63–83)	75	(66–82)	0.80
Male sex, *n* (%)	920	(64.1)	490	(65.5)	0.50
Body mass index, kg/m^2^, median (IQR)	26.8	(24.1–30.8)	26.1	(23.4–30.7)	0.08
SBP, mmHg, median (IQR)	144	(128–166)	140	(120–160)	**0.01**
DBP, mmHg, median (IQR)	80	(70–91)	74	(65–87)	**0.01**
Heart rate, bpm, median (IQR)	80	(69–96)	80	(68–94)	0.13
Medical history, *n* (%)					
Coronary artery disease	500	(34.8)	396	(52.9)	**0.01**
Prior myocardial infarction	285	(19.8)	236	(31.6)	**0.01**
Prior PCI	330	(23.0)	282	(37.7)	**0.01**
Prior CABG	125	(8.7)	89	(11.9)	**0.02**
Prior valvular surgery	52	(3.6)	44	(5.9)	**0.01**
Congestive heart failure	317	(22.1)	424	(56.7)	**0.01**
Decompensated heart failure < 12 months	0	(0.0)	238	(31.8)	**0.01**
Prior ICD	22	(1.5)	20	(2.7)	0.07
Prior sICD	3	(0.2)	6	(0.8)	**0.04**
Prior CRT-D	16	(1.1)	16	(2.1)	0.06
Prior Pacemaker	108	(7.5)	92	(12.3)	**0.01**
Chronic kidney disease	343	(23.9)	336	(44.9)	**0.01**
Peripheral artery disease	126	(8.8)	127	(17.0)	**0.01**
Stroke	193	(13.4)	138	(18.4)	**0.01**
Liver cirrhosis	28	(1.9)	19	(2.5)	0.37
Malignancy	158	(11.0)	177	(23.7)	**0.01**
COPD	139	(9.7)	124	(16.6)	**0.01**
Cardiovascular risk factors, *n* (%)					
Arterial hypertension	1091	(76.0)	611	(81.7)	**0.01**
Diabetes mellitus	512	(35.7)	287	(38.4)	0.21
Hyperlipidemia	429	(29.9)	233	(31.1)	0.54
Smoking	515	(35.9)	281	(37.6)	0.43
Current	291	(20.3)	115	(15.4)	**0.01**
Former	224	(15.6)	166	(22.2)	**0.01**
Family history	140	(9.7)	61	(8.2)	0.22
Comorbidities at index hospitalization, *n* (%)					
Acute coronary syndrome					
Unstable angina	64	(4.5)	35	(4.7)	0.81
STEMI	154	(10.7)	22	(2.9)	**0.01**
NSTEMI	211	(14.7)	63	(8.4)	**0.01**
Acute decompensated heart failure	261	(18.2)	223	(29.8)	**0.01**
Cardiogenic shock	36	(2.5)	17	(2.3)	0.74
Atrial fibrillation	542	(37.7)	374	(50.0)	**0.01**
Cardiopulmonary resuscitation	37	(2.6)	16	(2.1)	0.53
Out-of-hospital	17	(1.2)	5	(0.7)	0.25
In-hospital	20	(1.4)	11	(1.5)	0.88
Stroke	248	(17.3)	50	(6.7)	**0.01**
Medication on admission, *n* (%)					
ACE-inhibitor	482	(33.6)	293	(39.2)	**0.01**
ARB	309	(21.5)	180	(24.1)	0.18
Beta-blocker	714	(49.7)	520	(69.5)	**0.01**
Aldosterone antagonist	98	(6.8)	108	(14.4)	**0.01**
ARNI	4	(0.3)	15	(2.0)	**0.01**
SGLT2-inhibitor	24	(1.7)	21	(2.8)	0.08
Loop diuretics	407	(28.3)	414	(55.3)	**0.01**
Statin	570	(39.7)	415	(55.5)	**0.01**
ASA	454	(31.6)	281	(37.6)	**0.01**
P2Y12-inhibitor	86	(6.0)	125	(16.7)	**0.01**
DOAC	270	(18.8)	250	(33.4)	**0.01**
Vitamin K antagonist	109	(7.6)	76	(10.2)	**0.04**

Bold type indicates statistical significance

*ACE* angiotensin-converting enzyme; *ARB* angiotensin receptor blocker; *ARNI* angiotensin receptor neprilysin inhibitor; *ASA* acetylsalicylic acid; *CABG* coronary artery bypass grafting; *CKD* chronic kidney disease; *COPD* chronic obstructive pulmonary disease; *CRT-D* cardiac resynchronization therapy with defibrillator; *DBP* diastolic blood pressure; *DOAC* directly acting oral anticoagulant; *IQR* interquartile range; *(N)STEMI* non-ST-segment elevation myocardial infarction; *SBP* systolic blood pressure; *SGLT2* sodium glucose linked transporter 2; *(s) ICD* (subcutaneous) implantable cardioverter defibrillator

Level of significance *p* ≤ 0.05

Patients without a prior hospitalization had higher rates of concomitant acute coronary syndromes during index hospitalization, such as ST-segment elevation myocardial infarction (STEMI) (10.7% vs. 2.9%; *p* = 0.01), non-ST-segment elevation myocardial infarction (NSTEMI) (14.7% vs. 8.4%; *p* = 0.01) or stroke (17.3% vs. 6.7%; *p* = 0.01).

Table 2 displays data on HF-related characteristics and procedural data during index hospitalization. Patients with a prior hospitalization had higher NYHA functional class (NYHA III 24.9% vs. 15.6%, NYHA IV 12.7% vs. 6.5%; *p* = 0.01), alongside with higher rates of moderate to severe tricuspid (20.3% vs. 13.4%; *p* = 0.01), mitral (12.7% vs. 11.6%; *p* = 0.01) and aortic (5.1% vs. 3.2%; *p* = 0.03) regurgitation. With regard to baseline laboratory values, patients with a prior hospitalization had higher creatinine (median 1.19 mg/dL vs. 1.03 mg/dL; *p* = 0.01), C-reactive protein (CRP) (median 14.7 mg/L vs. 12.7 mg/L; *p* = 0.05) and NT-proBNP levels (3323 pg/mL vs. 2375 pg/mL; *p* = 0.01), but lower hemoglobin (median 11.4 mg/dL vs. 12.8 mg/dL; *p* = 0.01) and white blood cell counts (median 7.86 × 10⁹/L vs. 8.38 × 10⁹/L; *p* = 0.01) on index hospital admission. Furthermore, patients with a prior hospitalization were more commonly discharged on aldosterone antagonists (19.5% vs. 11.2%; *p* = 0.01), angiotensin receptor–neprilysin inhibitors (2.2% vs 0.6%; *p* = 0.01), loop diuretics (62.8% vs. 40.8%; *p* = 0.01), digitalis (6.4% vs. 4.1%; *p* = 0.02), amiodarone (4.6% vs. 1.8%; *p* = 0.01), vitamin k antagonists (9.3% vs. 6.0%; *p* = 0.01 and direct oral anticoagulants (DOAC) (37.9% vs. 30.0%; *p* = 0.01) compared to patients without prior hospitalization < 12 months.

**Table 2:** Heart failure-related and procedural data

	No prior hospitalization (*n* = 1436)	Prior hospitalization (*n* = 748)	*p* value
Heart failure etiology, *n* (%)			
Ischemic cardiomyopathy	823 (57.3)	435 (58.2)	**0.01**
Non-ischemic cardiomyopathy	80 (5.6)	69 (9.2)	
Hypertensive cardiomyopathy	127 (8.8)	51 (6.8)	
Congenital heart disease	2 (0.1)	2 (0.3)	
Valvular heart disease	49 (3.4)	47 (6.3)	
Tachycardia associated	66 (4.6)	24 (3.2)	
Tachymyopathy	23 (1.6)	15 (2.0)	
Pacemaker-induced cardiomyopathy	12 (0.8)	7 (0.9)	
Unknown	254 (17.7)	98 (13.1)	
NYHA functional class, *n* (%)			
I/II	1118 (77.9)	467 (62.4)	**0.01**
III	224 (15.6)	186 (24.9)	
IV	94 (6.5)	95 (12.7)	
Echocardiographic data			
LVEF, %, median (IQR)	45 (45–47)	45 (45–47)	0.72
IVSd, median (IQR)	12 (10–13)	12 (11–13)	0.35
LVEDD, mm, median (IQR)	49 (44–53)	49 (44–54)	0.21
TAPSE, mm, median (IQR)	20 (17–23)	20 (17–23)	0.08
LA diameter, mm, median (IQR)	41 (36–47)	43 (38–49)	**0.01**
LA surface, cm^2^, median (IQR)	21 (17–25)	23 (19–27)	**0.01**
E/A, median (IQR)	0.8 (0.6–1.2)	0.8 (0.7–1.2)	0.14
E/E′, median (IQR)	9.0 (6.5–13.5)	10.0 (6.3–14.3)	0.42
Diastolic dysfunction, *n* (%)	1045 (72.8)	529 (70.7)	0.31
Moderate–severe aortic stenosis, *n* (%)	141 (9.8)	73 (9.8)	0.97
Moderate–severe aortic regurgitation, *n* (%)	46 (3.2)	38 (5.1)	**0.03**
Moderate–severe mitral regurgitation, *n* (%)	167 (11.6)	95 (12.7)	0.47
Moderate–severe tricuspid regurgitation, *n* (%)	192 (13.4)	152 (20.3)	**0.01**
Coronary angiography, *n* (%)	652 (45.4)	248 (33.2)	**0.01**
No evidence of coronary artery disease	127 (19.5)	48 (19.4)	0.30
1-vessel disease	128 (19.6)	38 (15.3)	
2-vessel disease	142 (21.8)	50 (20.2)	
3-vessel disease	255 (39.1)	112 (45.2)	
CABG	42 (6.4)	31 (12.5)	**0.01**
Chronic total occlusion	81 (12.4)	32 (12.9)	0.85
PCI, *n* (%)	367 (56.3)	114 (46.0)	**0.01**
Sent to CABG, *n* (%)	39 (6.0)	12 (4.8)	0.51
Baseline laboratory values, median (IQR)			
Potassium, mmol/L	3.9 (3.6–4.2)	3.9 (3.6–4.2)	0.59
Sodium, mmol/L	139 (137–141)	139 (137–141)	0.33
Creatinine, mg/dL	1.03 (0.84–1.33)	1.19 (0.93–1.76)	**0.01**
eGFR, mL/min/1.73 m^2^	69 (50–89)	57 (36–79)	**0.01**
Hemoglobin, g/dL	12.8 (10.9–14.2)	11.4 (9.7–13.2)	**0.01**
WBC count, × 10^9^/L	8.38 (6.67–10.21)	7.86 (6.15–9.86)	**0.01**
Platelet count, × 10^9^/L	225 (180–282)	230 (176–294)	0.43
HbA1c, %	5.9 (5.5–6.8)	5.9 (5.5–6.8)	0.78
LDL-cholesterol, mg/dL	102 (77–129)	90 (67–117)	**0.01**
HDL-cholesterol, mg/dL	42 (34–52)	42 (34–52)	0.80
C-reactive protein, mg/L	12.7 (3.0–43.3)	14.7 (4.3–46.0)	**0.05**
NT-pro BNP, pg/mL	2375 (802–5680)	3323 (1544–7919)	**0.01**
Cardiac troponin I, µg/L	0.03 (0.02–0.25)	0.03 (0.02–0.12)	0.07
Medication at discharge, *n* (%)			
ACE-inhibitor	725 (52.1)	333 (46.4)	**0.01**
ARB	320 (23.0)	179 (25.0)	0.31
Beta-blocker	1066 (76.6)	569 (79.4)	0.15
Aldosterone antagonist	156 (11.2)	140 (19.5)	**0.01**
ARNI	9 (0.6)	16 (2.2)	**0.01**
SGLT2-inhibitor	59 (4.2)	25 (3.5)	0.40
Loop diuretics	568 (40.8)	450 (62.8)	**0.01**
Statin	974 (70.0)	468 (65.3)	**0.03**
Digitalis	57 (4.1)	46 (6.4)	**0.02**
Amiodarone	25 (1.8)	33 (4.6)	**0.01**
ASA	748 (53.7)	315 (43.9)	**0.01**
P2Y12-inhibitor	465 (33.4)	203 (28.3)	**0.02**
DOAC	418 (30.0)	272 (37.9)	**0.01**
Vitamin k antagonist	83 (6.0)	67 (9.3)	**0.01**

Bold type indicates statistical significance

*ACE* angiotensin-converting enzyme; *ARB* angiotensin receptor blocker; *ARNI* angiotensin receptor neprilysin inhibitor; *ASA* acetylsalicylic acid; *CABG* coronary artery bypass grafting; *DOAC* directly acting oral anticoagulant; *eGFR* estimated glomerular filtration rate; *HbA1c* glycated hemoglobin; *HDL* high-density lipoprotein; *IQR* interquartile range; *IVSd* interventricular septal end diastole; *LA* left atrial; *LDL* low-density lipoprotein; *LVEDD* left ventricular end-diastolic diameter; *LVEF* left ventricular ejection fraction; *NT-pro BNP* aminoterminal pro-B-type natriuretic peptide; *NYHA* New York Heart Association; *PCI* percutaneous coronary intervention; *TAPSE* tricuspid annular plane systolic excursion; *WBC* white blood cells

Level of significance *p* ≤ 0.05

### Prognostic impact of prior hospitalizations in patients with HFmrEF

**Fig. 2:**
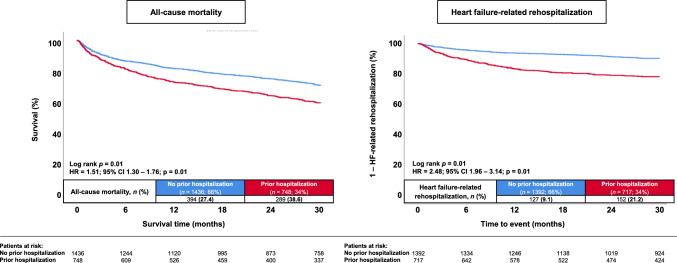
Kaplan–Meier analyses investigating the outcomes of patients with and without a prior hospitalization < 12 months in HFmrEF regarding the risk of all-cause mortality (left panel) and HF-related rehospitalization (right panel) at 30 months

At 30 months, the risk of all-cause mortality was significantly higher in patients with at least one prior hospitalization < 12 months before index hospitalization (38.6% vs. 27.4%; HR = 1.51; 95% CI 1.30–1.76; *p* = 0.01; log-rank *p* = 0.01) (Table 3, Fig. 2, left panel). In line, the risk of all-cause mortality at 12 months was already increased in patients with a prior hospitalization (27.3% vs. 18.2%; HR = 1.57; 95% CI 1.30–1.89; *p* = 0.01). A prior hospitalization was furthermore associated with an increased risk of HF-related rehospitalization at 12 (16.6% vs. 6.1%; HR = 2.86; 95% CI 2.17–3.79; *p* = 0.01) and 30 months (21.2% vs. 9.1%; HR = 2.48; 95% CI 1.96–3.14; *p* = 0.01; log-rank *p* = 0.01) (Table 3, Fig. 2, right panel). In line, a higher proportion of patients with a prior hospitalization experienced cardiac rehospitalizations at 30 months (HR = 1.93; 95% CI 1.61–2.31; *p* = 0.01), alongside with higher rates of AMI at 30 months (HR = 2.50; 95% CI 1.53–4.10; *p* = 0.01). Finally, the risk of MACCE at 30 months was increased in patients with at least one prior hospitalization (HR = 1.53; 95% CI 1.34–1.76; *p* = 0.01).

**Table 3:** Follow-up data, primary and secondary endpoints

	No prior hospitalization (*n* = 1436)	Prior hospitalization (*n* = 748)	HR	95% CI	*p* value
Primary endpoint, *n* (%)					
All-cause mortality, at 30 months	394 (27.4)	289 (38.6)	1.51	(1.30–1.76)	**0.01**
Secondary endpoints, *n* (%)					
All-cause mortality, in-hospital	44 (3.1)	31 (4.1)	0.73*	(0.46–1.17)*	0.19*
Cardiac mortality, in-hospital	15 (1.0)	9 (1.2)	0.87*	(0.38–2.0)*	0.74*
Non-cardiac mortality, in-hospital	29 (2.0)	22 (2.9)	0.68*	(0.39–1.19)*	0.18*
All-cause mortality, at 12 months	262 (18.2)	204 (27.3)	1.57	(1.30–1.89)	**0.01**
Heart-failure related rehospitalization, at 12 months	85 (6.1)	119 (16.6)	2.86	(2.17–3.79)	**0.01**
Heart-failure related rehospitalization, at 30 months	127 (9.1)	152 (21.2)	2.48	(1.96–3.14)	**0.01**
Cardiac rehospitalization, at 30 months	241 (17.3)	221 (30.8)	1.93	(1.61–2.31)	**0.01**
Coronary revascularization, at 30 months	83 (6.0)	59 (8.2)	1.38	(0.99–1.93)	0.06
Acute myocardial infarction, at 30 months	28 (2.0)	36 (5.0)	2.50	(1.53–4.10)	**0.01**
Stroke, at 30 months	34 (2.4)	23 (3.2)	1.30	(0.76–2.20)	0.34
MACCE, at 30 months	487 (33.9)	354 (47.3)	1.53	(1.34–1.76)	**0.01**
Follow-up data, median (IQR)					
Hospitalization time, days	8 (5–14)	9 (5–17)	–	–	**0.05**
ICU time, days	0 (0–1)	0 (0–0)	–	–	**0.01**
Follow-up time, days	946 (431–1709)	796 (286–1506)	–	–	**0.01**

Bold type indicates statistical significance

*CI* confidence interval; *HR* hazard ratio; *ICU* intensive care unit; *MACCE* major adverse cardiac and cerebrovascular events

Level of significance *p* ≤ 0.05

*An odds ratio with the corresponding 95% CI and *p*-value was provided for the endpoints of in-hospital mortality, accounting for the variance in hospitalization time

After multivariable adjustment for important baseline characteristics and comorbidities (Table 4), the presence of at least one prior hospitalization < 12 months *p* was independently associated with an increased risk of 30-months all-cause mortality (HR = 1.22; 95% CI 1.03 - 1.45; p = 0.02). Further significant predictors of 30-month all-cause mortality included age (HR = 1.42; 95% CI 1.32–1.53; *p* = 0.01), the presence of CKD (HR = 1.59; 95% CI 1.35–1.88; *p* = 0.01), acute decompensated heart failure (ADHF) (HR = 1.36; 95% CI 1.11–1.67; *p* = 0.01) and right ventricular dysfunction (HR = 1.39; 95% CI 1.17–1.66; *p* = 0.01). In line, the presence of concomitant CKD (HR = 1.55; 95% CI 1.19–2.02; *p* = 0.01), prior congestive HF (HR = 1.58; 95% CI 1.20–2.07; *p* = 0.01), diabetes mellitus (HR = 1.36; 95% CI 1.06–1.73; *p* = 0.02), ADHF (HR = 1.45; 95% CI 1.07–1.96; *p* = 0.02), atrial fibrillation (HR = 1.75; 95% CI 1.34–2.28; *p* = 0.01), and the presence of a prior hospitalization < 12 months (HR = 1.55; 95% CI 1.20–2.01; *p* = 0.01) predicted the risk of HF-related rehospitalization at 30 months.

**Table 4:** Multivariable Cox regression analyses with regard to 30-month all-cause mortality and heart failure-related re-hospitalization within the entire study cohort

Variables	All-cause mortality					
	Univariable			Multivariable		
	HR	95% CI	*p* value	HR	95% CI	*p* value
Age (per decade increase)	1.62	1.51–1.74	**0.01**	1.42	1.32–1.53	**0.01**
Male sex	0.93	0.80–1.09	0.37	–	–	–
Prior congestive heart failure	1.55	1.33–1.80	**0.01**	1.08	0.91–1.29	0.39
Chronic kidney disease	2.52	2.17–2.93	**0.01**	1.59	1.35–1.88	**0.01**
Diabetes mellitus	1.28	1.10–1.49	**0.01**	1.04	0.89–1.22	0.59
Acute myocardial infarction	0.62	0.50–0.77	**0.01**	1.01	0.80–1.29	0.91
Acute decompensated heart failure	2.27	1.94–2.66	**0.01**	1.36	1.11–1.67	**0.01**
Atrial fibrillation	1.86	1.60–2.16	**0.01**	1.12	0.95–1.32	0.19
Ischemic cardiomyopathy	0.79	0.68–0.91	**0.01**	0.72	0.61–0.86	**0.01**
LVEF (per 1% increase)	0.97	0.94–1.01	0.12	–	–	–
Diastolic dysfunction	0.93	0.79–1.10	0.37	–	–	–
NYHA functional class	1.35	1.26–1.45	**0.01**	1.03	0.94–1.12	0.58
Right ventricular dysfunction*	1.86	1.57–2.20	**0.01**	1.39	1.17–1.66	**0.01**
Moderate–severe aortic stenosis	1.96	1.59–2.41	**0.01**	1.24	1.00–1.54	**0.05**
Moderate–severe mitral regurgitation	1.83	1.50–2.22	**0.01**	1.16	0.94–1.42	0.16
Prior hospitalization < 12months	1.51	1.30–1.76	**0.01**	1.22	1.03–1.45	**0.02**
Prior internal medicine vs. surgical admission	0.82	0.63–1.08	0.16	–	–	–
Prior heart failure-related admission	1.27	0.85–1.90	0.24	–	–	–
Prior emergency admission	1.36	0.93–1.98	0.12	–	–	–
Prior elective admission (reference group)						

Variables	Heart failure-related re-hospitalization					
	Univariable			Multivariable		
	HR	95% CI	*p* value	HR	95% CI	*p* value
Age (per decade increase)	1.35	1.22–1.48	**0.01**	1.02	0.91–1.14	0.76
Male sex	0.76	0.60–0.97	0.03	0.83	0.65–1.06	0.14
Prior congestive heart failure	2.97	2.34–3.78	**0.01**	1.58	1.20–2.07	**0.01**
Chronic kidney disease	2.97	2.34–3.76	**0.01**	1.55	1.19–2.02	**0.01**
Diabetes mellitus	1.70	1.34–2.14	**0.01**	1.36	1.06–1.73	**0.02**
Acute myocardial infarction	0.55	0.39–0.78	**0.01**	0.79	0.54–1.15	0.22
Acute decompensated heart failure	3.25	2.57–4.12	**0.01**	1.45	1.07–1.96	**0.02**
Atrial fibrillation	2.59	2.03–3.30	**0.01**	1.75	1.34–2.28	**0.01**
Ischemic cardiomyopathy	1.35	1.06–1.73	**0.02**	1.38	1.06–1.81	**0.02**
LVEF (per 1% increase)	1.02	0.96–1.08	0.55	–	–	–
Diastolic dysfunction	0.98	0.76–1.28	0.89	–	–	–
NYHA functional class	1.74	1.56–1.93	**0.01**	1.24	1.07–1.42	**0.01**
Right ventricular dysfunction*	1.70	1.30–2.20	**0.01**	1.05	0.80–1.39	0.71
Moderate–severe aortic stenosis	2.06	1.51–2.82	**0.01**	1.60	1.15–2.23	**0.01**
Moderate–severe mitral regurgitation	2.06	1.54–2.77	**0.01**	1.47	1.09–1.99	**0.01**
Prior hospitalization < 12months	2.48	1.96–3.14	**0.01**	1.55	1.20–2.01	**0.01**
Prior internal medicine vs. surgical admission	1.47	0.98–2.24	0.07	0.89	0.65–1.22	0.47
Prior heart failure-related admission	2.24	1.26–3.98	**0.01**	0.97	0.66–1.44	0.89
Prior emergency admission	1.34	0.75–2.37	0.32	–	–	–
Prior elective admission	(reference group)					

Multivariable risk prediction models were in addition performed with regard to the prognostic impact of the department and type of prior hospital admission

Bold type indicates statistical significance

*ADHF* acute decompensated heart failure; *CI* confidence interval; *CKD* chronic kidney disease; *DM* diabetes mellitus; *HR* hazard ratio; *LVEF* left ventricular ejection fraction; *NYHA* New York Heart Association; *TAPSE* tricuspid annular plane systolic excursion

Level of significance *p* ≤ 0.05

*Right ventricular dysfunction was defined as TAPSE < 17 mm

### Prognostic impact of the admission department of prior hospitalizations

**Fig. 3:**
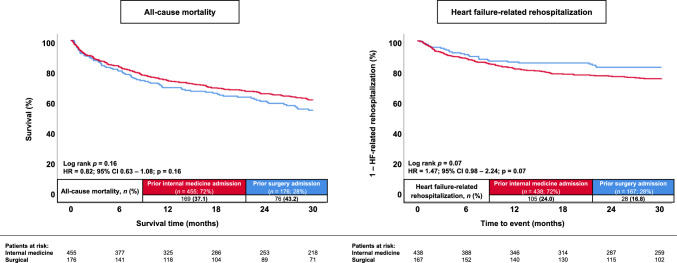
Kaplan–Meier analyses investigating the outcomes of patients with prior hospitalization in HFmrEF stratified by a prior admission to a department of internal medicine or surgical department with regard to the risk of all-cause mortality (left panel) and HF-related rehospitalization (right panel) at 30 months

Patients with a previous admission to a surgical department were not statistically significant associated with a higher risk of 30-months all-cause mortality (43.2% vs. 37.1%; HR = 0.82; 95% CI 0.63–1.08; *p* = 0.16; log-rank *p* = 0.16) (Fig. 3, left panel). Compared to patients admitted to an internal medicine department, a previous hospitalization was associated with numerically higher rates of rehospitalization for worsening HF, although this association did not reach statistical significance (16.8% vs. 24.0%; HR = 1.47; 95% CI 0.98–2.24; *p* = 0.07; Log-rank *p* = 0.07) (Fig. 3, right panel). The department of admission in patients with prior hospitalization was not associated with the risk of HF-related rehospitalization after multivariable adjustment (HR = 0.89; 95% CI 0.65–1.22; *p* = 0.47) (Table 4).

### Prognostic impact of the type of prior hospitalization

**Fig. 4:**
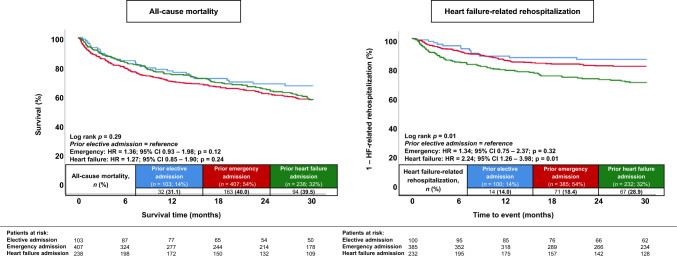
Kaplan–Meier analyses investigating the outcomes of patients with the prior hospitalization in HFmrEF stratified by the type of prior admission with regard to the risk of all-cause mortality (left panel) and HF-related rehospitalization (right panel)

Finally, the type of prior hospitalization (i.e., elective, emergency non-HF or HF-related) was not significantly associated with the risk of 30-months all-cause mortality (log-rank *p* = 0.29) (emergency: HR = 1.36; 95% CI 0.93–1.98; *p* = 0.12; HF-related: HR = 1.27; 95% CI 0.85–1.90; *p* = 0.24; elective = reference group) (Fig. 4, left panel). However, patients with a prior hospitalization for HF had an increased risk of HF-related rehospitalization rates at 30 months (log-rank *p* = 0.01) (emergency: HR = 1.34; 95% CI 0.75–2.37; *p* = 0.32; HF-related: HR = 2.24; 95% CI 1.26–3.98; *p* = 0.01; elective = reference group) (Fig. 4, right panel). After multivariable adjustment, the type of prior hospitalization (i.e., HF-related, emergency non-HF-related) was not associated with the risk of HF-related rehospitalization in patients with HFmrEF (HR = 0.97; 95% CI 0.66–1.44; *p* = 0.89) (Table 4).

## Discussion

The aim of the present study was to investigate the prognostic impact of a prior hospitalization < 12 months in a large cohort of consecutive patients hospitalized with HFmrEF. Patients with a prior hospitalization presented with a higher burden of cardiovascular and non-cardiovascular comorbidities. A prior hospitalization was independently associated with an increased risk of long-term all-cause mortality and HF-related rehospitalization. Furthermore, a prior hospitalization was associated with higher rates of AMI, coronary revascularization and MACCE at 30 months. However, the risk of long-term all-cause mortality in patients with prior hospitalizations was not significantly affected by the department of previous hospital admission, as well as by the type of prior hospitalization (i.e., elective, emergency non-HF or HF-related).

Previous studies investigating the prognosis of HF patients have reported heterogenous estimates of mortality and rehospitalization rates for HFpEF and HFrEF [11, 20–32]. Whereas some studies suggested a similar risk of all-cause mortality in HF patients regardless of LVEF [20–22, 26, 32], others suggested a lower risk of all-cause mortality in HFpEF compared to HFrEF patients [23–25, 29–31]. This inconsistency may rely on pre-selected inclusion criteria, such as the inclusion of inpatients or outpatients, as well as on the inclusion of acute vs. chronic HF [20–32]. The CHARM analysis demonstrated an inverse relationship between time from HF hospitalization to trial randomization with regard to cardiovascular death or HF-related hospitalization for both HFpEF and HFrEF. Although prior HF hospitalizations increased the risks similarly across the EF spectrum, patients with low LVEF consistently exhibited higher event rates compared to those with HFpEF [12]. The trend of declining event rates including death and HF-realted rehospitalization in cardiovascular trials over time may relate to the implementation of proven interventions in clinical care, necessitating larger patient numbers to detect significant differences [33–35]. Bello et al*.* suggested that a recent HF hospitalization can identify high-risk patients in patients with HFpEF, being associated with similar risk of death or HF-related rehospitalization compared to HFrEF patients without prior hospitalization [12].

Our findings for the HFmrEF cohort are in line with those reported for HFrEF and HFpEF. Patients with HFmrEF hospitalized in the past 12 months were associated with impaired prognosis, akin to the elevated event rates observed in HFrEF. This observation aligns with recent studies [7, 8, 16, 36, 37], which indicate that HFmrEF and HFrEF share clinical characteristics and therapeutic responses including response to HF-related pharmacotherapies [38]. In addition, HFmrEF patients exhibited a high burden of comorbidities and severe illness, paralleling the profile of HFpEF patients with recent hospitalizations [39, 40]. These findings underscore the intermediate position of HFmrEF. But integrating later published data such as those from Malik et al*.* must consider that the “preserved” LVEF group in the CHARM-programme by Swedberg et al*.* [41] was defined as an LVEF > 40%. Today, this definition encompasses the HFmrEF cohort which may explain the observed similarities in recent hospitalizations between the HFpEF and HFmrEF cohorts.

Furthermore, the management of HF patients by cardiologists vs internists may significantly affect patients’ outcomes. Cardiologists tend to treat younger, predominantly male patients with more severe cardiac dysfunction, specifically HFrEF, accompanied by lower burden with comorbidities, adhering more closely to HF guidelines such as the use of angiotensin-converting enzyme (ACE)-inhibitors and beta-blockers, resulting in lower 9-month and 6-month cardiac-related mortality rates [42–44]. Conversely, internists manage older, often female patients with a higher burden of comorbidities including hypertension, diabetes, COPD, previous stroke/transient ischemic attack, pulmonary congestion, and peripheral edema, which adversely impacts prognosis [42, 45]. High-volume hospitals and those with greater cardiology involvement show better outcomes, including lower 30-day mortality, readmission rates, and hospitalization costs, emphasizing the importance of specialist experience [46]. While cardiologist care is associated with higher costs and resource use, it results in improved adherence to guidelines and reduced mortality and morbidity [44, 47]. Despite similar readmission and short-term mortality rates across specialties, the increased comorbidity burden managed by internists poses a significant prognostic challenge, necessitating tailored care strategies [48, 49]. However, those studies did not investigate the risk of all-cause mortality stratified by the departments of prior hospital admission. Within the present study however, the prognosis of patients previously admitted to an internal or surgical department did not significantly differ. This may be in line with an overall high burden of non-cardiovascular comorbidities among patients with a previous hospitalization, as demonstrated within the present study (i.e., CKD: 44.9%, malignancy: 23.7%, COPD: 16.6%).

This finding aligns with previous studies highlighting that the management of non-cardiac comorbidities, such as diabetes, renal disease, and obesity, significantly impacts patient outcomes in HF across different EF groups. The study by Lindberg et al. [50] demonstrated that specialty care is associated with better survival rates, particularly for patients with HFrEF or borderline ejection fraction (HFbEF), research by Mentz et al. [51] emphasized the detrimental effects of comorbidities on both HFrEF and HFpEF patient outcomes, underlining the complexity of managing HF in the presence of multiple chronic conditions.

## Study limitations

Given the retrospective and single-center nature of the study, measured and unmeasured confounding factors may still be present, even after multivariable adjustments. This may limit the generalizability of the findings. In addition, HF-related and cardiac rehospitalizations were only assessed at our institution, which may not fully capture the overall rehospitalization rates. Data on patients' perfusion status and fluid management were not included in the study. Information regarding causes of death occurring beyond the index hospitalization was also unavailable.

## Conclusions

This study suggests that a prior hospitalization < 12 months is common in HFmrEF and significantly impacts long-term prognosis. Patients with a prior hospitalization exhibited a higher burden of cardiovascular and non-cardiovascular comorbidities. However, the admission department and type of prior hospitalization (i.e., elective, emergency or HF-related) did not significantly affect the risk of long-term all-cause mortality. The study therefore highlights the importance to consider prior hospitalizations as an important independent risk factor in patients with HFmrEF.
